# Pharmacodynamic Modeling of Cinacalcet in Secondary Hyperparathyroidism: Efficacy and Influencing Factors Analysis

**DOI:** 10.1210/jendso/bvaf021

**Published:** 2025-03-27

**Authors:** Zhizhou Wang, Yexuan Wang, Shun Han, Peixian Chen, Ruo Wu, Chuyao Fang, Junjie Cheng, Yujiao Wu, Tingting Guo, Xin Wen, Lujin Li

**Affiliations:** Department of Pharmacy, Jinshan Hospital, Fudan University, Shanghai, 201508 Shanghai, China; Center for Drug Clinical Research, Shanghai University of Traditional Chinese Medicine, Shanghai, 201203 Shanghai, China; Department of Orthopedic Surgery, Sun Yat-sen Memorial Hospital, Sun Yat-Sen University, Guangzhou, 510120 Guangdong, China; Department of Urology, Sun Yat-sen Memorial Hospital, Sun Yat-sen University, Guangzhou, 510120 Guangdong, China; Department of Cardiovascular Medicine, The Eighth Affiliated Hospital, Sun Yat-sen University, Shenzhen, 518033 Guangdong, China; Department of Radiation Oncology, Nanfang Hospital, Southern Medical University, Guangzhou, 510515 Guangdong, China; Department of Student Office, The Second Clinical College of Southern Medical University, Guangzhou, 510515 Guangdong, China; Department of Student Office, The Second Clinical College of Southern Medical University, Guangzhou, 510515 Guangdong, China; Department of Nephrology, Zhujiang Hospital, Southern Medical University, Guangzhou, Guangdong 501280, China; Department of Clinical Research Centre, Zhujiang Hospital, Southern Medical University, Guangzhou, Guangdong 501280, China; Center for Drug Clinical Research, Shanghai University of Traditional Chinese Medicine, Shanghai, 201203 Shanghai, China

**Keywords:** secondary hyperparathyroidism, cinacalcet, model based meta-analysis, serum parathyroid hormone, serum calcium, serum phosphorus

## Abstract

**Context:**

Cinacalcet, the first FDA-approved calcimimetic agent for treating secondary hyperparathyroidism (SHPT), has unclear factors influencing its therapeutic efficacy in clinical practice.

**Objective:**

To establish a pharmacodynamic model for cinacalcet use in SHPT, analyze drug effect distribution and influencing factors, and determine optimal treatment strategy.

**Methods:**

We searched public databases for randomized trials on cinacalcet for SHPT, modeling changes in serum parathyroid hormone (PTH), calcium, and phosphorus postintervention. Key pharmacodynamic parameters and influencing factors were identified, with subgroup analysis for factors not in the covariate model. We also compared cinacalcet efficacy between United States/European Union (30-180 mg) and Asia (25-100 mg) dosage ranges.

**Results:**

Twenty-six studies (4242 subjects) were analyzed. Covariate analysis showed increasing PTH baseline and vitamin D use proportionally affected PTH and calcium decrease. Postintervention, maximum effects were observed with onset times of 0.46, 0.15, and 0.29 months. Subgroup analysis showed factors such as dialysis time, baseline calcium and phosphorus, phosphate binder use, gender proportion, patient ethnicity, blinding, and age influenced PTH, calcium, and phosphorus decrease. The efficacy of cinacalcet at a dosage of 25 to 100 mg in Asian populations was comparable to that observed at a dose range of 30 to 180 mg in Western populations, suggesting that reducing the therapeutic dose of cinacalcet may potentially yield a better benefit–risk ratio.

**Conclusion:**

We established a pharmacokinetic model for cinacalcet in SHPT treatment, providing crucial data for identifying effective patient populations and optimizing treatment strategies.

Chronic kidney disease, marked by irreversible progression, represents a major global health issue due to high prevalence and mortality rates [1, 2]. Secondary hyperparathyroidism (SHPT) is a common, serious complication in patients with chronic kidney disease, characterized by increased parathyroid hormone (PTH) levels, parathyroid hyperplasia, and disruptions in bone mineral metabolism [3-5]. Elevated PTH secretion heightens the risk for renal osteodystrophy, and vascular and soft tissue calcifications, thereby increasing mortality [6]. The 2017 Kidney Disease: Improving Global Outcomes (KDIGO) guidelines highlight the importance of monitoring serum phosphorus, calcium, and PTH levels [4]. Traditional treatments like calcium salts and vitamin D derivatives are limited in simultaneously managing serum calcium and phosphorus levels and reducing PTH [7]. The introduction of calcimimetics has greatly expanded treatment options for SHPT.

Cinacalcet, the first FDA-approved calcimimetic, inhibits PTH secretion and cell proliferation by activating the calcium sensing receptor [8], thus preventing increases in serum calcium and phosphorus levels [9]. A meta-analysis [10] has shown that cinacalcet effectively reduces PTH levels in patients with SHPT and enhances the achievement of target biochemical markers, including serum calcium and phosphorus. The current KDIGO guidelines recommend calcimimetics as first-line therapy for reducing PTH levels [4].

Despite the widespread use of cinacalcet, its practical application is still uncertain due to varying therapeutic efficacy reported across studies. A randomized controlled trial indicated that cinacalcet's efficacy was not dependent on disease severity or variations in vitamin D dosage [11]. Conversely, another study suggested higher effectiveness in patients with severe SHPT [12]. Factors such as baseline serum calcium and phosphorus levels, dialysis duration, and patient age may influence drug efficacy and require further study. Additionally, regional differences in cinacalcet dosing have been observed, with typical dosages ranging from 30 to 180 mg in the United States and European Union, while lower doses (25-100 mg) are used in Asia. A study by Fukagawa et al [13] highlighted that lower dosages effectively suppressed serum PTH levels in Japanese subjects, suggesting a need for further research to determine the suitability of lower cinacalcet dosages.

Model-based meta-analysis (MBMA) establishes a pharmacodynamic model facilitating quantitative comparisons of drug efficacy while mitigating trial heterogeneity effects [14, 15]. MBMA constructs comprehensive models, including time-course, dose–effect, and covariate models, aiding in the understanding of temporal and dose-related drug effects and influencing factors [16]. This methodology is crucial for guiding drug development strategies. In this study, we will utilize extensive literature data to establish an MBMA-based pharmacodynamic model for cinacalcet treatment of SHPT, elucidating its influencing factors and offering insights for rational clinical cinacalcet administration.

## Materials and Methods

### Research Criteria and Eligibility

We conducted a systematic search across PubMed, EMBASE, and the Cochrane Library for randomized controlled trials assessing the efficacy of cinacalcet in treating SHPT, completed on November 25, 2023. Details of the search strategy are available elsewhere (Form S1 [17]). The study follows the Preferred Reporting Items for Systematic Reviews and Meta-Analyses (PRISMA) guidelines. As the dataset for this study was derived from preexisting literature, the Ethics Committee of Zhujiang Hospital of Southern Medical University granted an exemption from ethics review. Inclusion and exclusion criteria are detailed elsewhere (Method S1 [17]).

### Data Extraction

Information such as literature characteristics, trial design characteristics, subjects’ characteristics, and clinical outcomes indicators (changes in serum PTH, calcium, and phosphorus concentrations) was extracted. The detailed data extraction method is described (see Method S1 [17]).

### Literature Quality Assessment

The quality of the literature was assessed using the Cochrane risk of bias criteria [18] by 2 researchers independently. Any differences were resolved through discussions with another researcher. The detailed evaluation strategies are described (see Method S2 [17]).

### Model Establishment and Evaluation

This study will develop a time-course model of serum PTH, calcium, and phosphorus levels in patients with SHPT following cinacalcet intervention. Efficacy variations across different trials will be analyzed using structural, random-effect, and covariate models. Methods for model construction and evaluation are detailed elsewhere (Method S3 [17]).

### Typical Effect Analysis

Based on the final pharmacodynamic model of cinacalcet, we can simulate temporal changes in serum PTH, calcium, and phosphorus levels at various time points postcinacalcet intervention in patients with SHPT, calculating the time to achieve 50% and 90% of their maximum effects. Additionally, if a covariate model influencing cinacalcet's pharmacodynamics is established, we will simulate typical effects on these biomarkers under varying covariate levels.

We will conduct subgroup analyses on factors such as the use of vitamin D compounds or phosphate binders, dialysis duration, proportion of White patients (as this is the only racial or ethnic group commonly reported in literature), age, gender, baseline serum calcium and phosphorus levels, and use of blinding, regardless of their inclusion in the final covariate model. These analyses aim to examine the impact of these factors on cinacalcet efficacy. Details are provided elsewhere (Method S4 [17]).

The KDIGO guidelines recommend a PTH target range of 2 to 9 times the upper normal limit (130-600 pg/mL), which this study will use to simulate the time to target achievement postcinacalcet treatment across different covariate levels. Dosing of cinacalcet varies between Asia (25-100 mg) and the United States/European Union (30-180 mg); this study will compare the efficacy of these dosages to assess the impact of dose variation on cinacalcet effectiveness.

### Software

Microsoft Excel (version 2019) software was utilized as the database entry template for data recording. Model estimation was performed using NONMEM 7.4 (ICON Development Solutions, USA), with the relevant code provided (see Method S5 [17]). The assessment of the quality of the literature was conducted using Review Manager (version 5.4, Nordic Cochrane Center, Copenhagen, Denmark). Model simulations, meta-analysis, and related plotting were carried out using R software (version 4.0.3, The R Foundation for Statistical Computing, Vienna, Austria).

## Results

### Characteristics of the Selected Studies

A total of 727 studies were retrieved, with 26 (3.6%) meeting the inclusion criteria, covering 4242 subjects (average age 55.7 years; 39.6% female, 60.4% male). Of these, 20 reported on PTH, 20 on serum calcium, and 17 on serum phosphorus. Baseline values ranged from 174.2 to 1281.4 pg/mL (median, 636 pg/mL) for PTH, 8.94 to 11.28 mg/dL (median, 9.73 mg/dL) for serum calcium, and 4.4 to 7.1 mg/dL (median, 5.75 mg/dL) for serum phosphorus. Screening processes, demographic characteristics, and quality assessments of the studies are detailed elsewhere (Figs. S1, S2, and Forms S2, S3 [17]).

### Modelling Results

Given the titrated dosing protocol of cinacalcet, where dosing escalates until the target effect is achieved, the clinical studies included primarily used dose ranges (30-180 mg), rather than fixed doses. This variability complicates the construction of a precise dose–effect model. To minimize the impact of dose heterogeneity, this study will focus on modeling and analyzing studies within the 30 to 180 mg cinacalcet dose range, exploring factors influencing its efficacy.

Covariate analysis identified that baseline PTH levels and the use of vitamin D compounds significantly affect the maximum effects (E_max_) of PTH and calcium, respectively (selection process in Fig. S3 [17]). No significant covariates were found for serum phosphorus time-course model parameters. Final model parameter estimates are presented in Table 1, with detailed results elsewhere (supplemental material [17]).

**Table 1.** Parameters estimation of the final model

Parameters	PTH		CA		P	
	Estimates (RSE%)	Bootstrap median (95% CI)	Estimates (RSE%)	Bootstrap median (95% CI)	Estimates (RSE%)	Bootstrap median (95% CI)
Pharmacodynamic parameters						
E_max_	308 (5.9)	310 (273, 350)	0.811 (4.0)	0.809 (0.733, 0.874)	0.524 (14.1)	0.539 (0.396, 0.677)
ET50	0.46 (18.0)	0.45 (0.307, 0.650)	—	—	—	—
K	—	—	4.64(13.9)	4.67 (3.14, 6.14)	2.4 (22.4)	2.2 (1.5, 2.9)
θ (baseline) on E_max_	0.503 (8.4)	0.508 (0.421, 0.626)	—	—	—	—
θ (VD) on E_max_	—	—	−0.00588 (20.7)	−0.00586 (−0.00826, −0.00274)	—	—
Interstudy variability						
ηE_max_, %	19.3 (21.1)	18.3 (9.5, 27.1)	12.1 (22.9)	10.8 (3.6, 16.8)	45.4 (26.7)	42.2 (18.9, 67.4)
ηET50, %	67.7 (17.5)	65.9 (36.6, 93.9)	—	—	—	—
ηK	—	—	—	—	0.763 (21.8)	0.726 (0.158, 1.074)
Residual error						
ɛ	1.463 (14.0)	1.453 (1.105, 1.825)	1.881 (13.7)	1.849 (1.360, 2.421)	1.597 (5.2)	1.572 (1.431, 1.713)

Abbreviations: θ, covariate correction factor; η, intertrail variability; ɛ, residual; CA, serum calcium; E_max_, theoretical maximum effect value; ET50, time to reach half of the maximum effect value; K, onset rate of the drug effect; P, serum phosphorus; PTH, parathyroid hormone; RSE, relative standard error.

**Figure 1.**
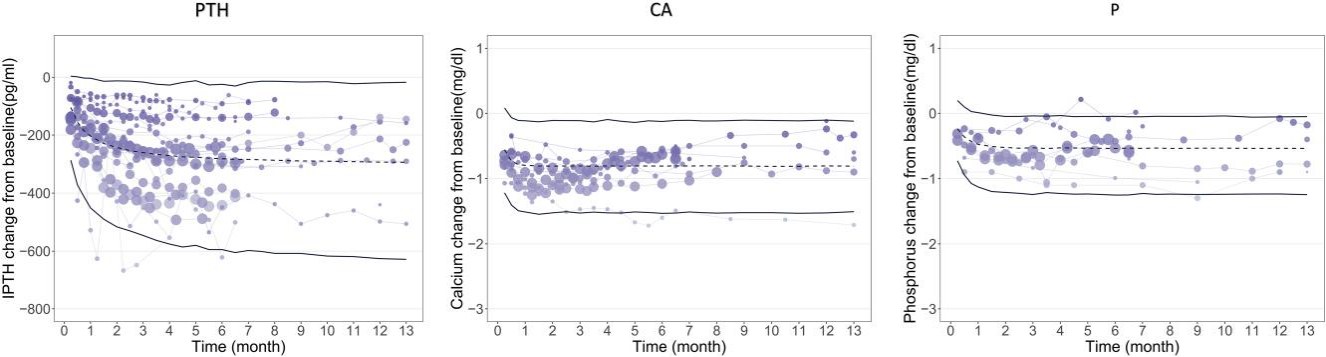
Visual predictive check for the final model of parathyroid hormone (PTH), serum calcium (CA), and serum phosphorus (P). Within the figure, the lines denote the 2.5th, 50th, and 97.5th percentiles of the drug effect as predicted by the model. The points represent observed values, with the size of each symbol being proportional to the sample size. Points connected by continuous lines originate from the same group.

Diagnostic plots confirm a good fit between the final model and the observed data (Fig. S4 [17]). Bootstrap parameter distributions closely match original estimates, indicating robust model parameter estimation (Table 1). The visual predictive check shows most observed values within the 95% CI, demonstrating strong model predictive accuracy (Fig. 1). Sensitivity analysis using leave-one-out cross-validation indicates minimal influence of individual studies on model parameters (Fig. S5 [17]).

### Typical Effects of PTH at Different Baseline Levels

**Figure 2.**
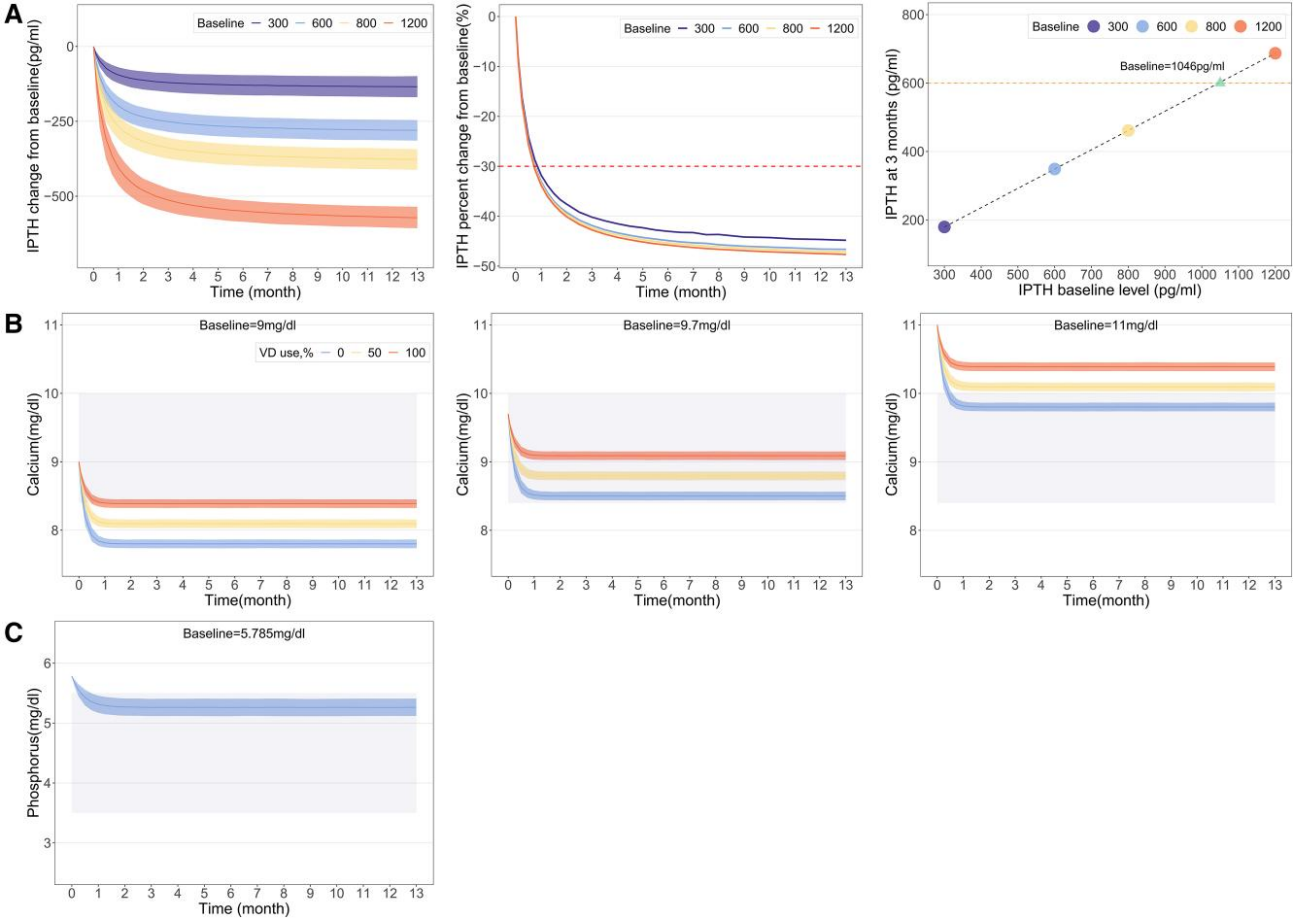
Model-estimated typical drug efficacy and 95% CI for parathyroid hormone (PTH) (A), serum calcium (B), and serum phosphorus (C) at different covariate levels. The solid lines denote the model-estimated typical effects, while the shaded areas correspond to their 95% CIs. (A) The red dashed line represents a 30% decrease from baseline, the orange dashed line indicates a PTH level of 600 pg/mL, which is approximately 9 times the upper limit of normal. (B) Different colors represent different proportions of patients on vitamin D compounds, and the rectangular shaded area delineates the target calcium range of 8.4 to 10 mg/dL. (C) The rectangular shaded area indicates the target phosphorus range of 3.5 to 5.5 mg/dL.

Using the final model, we simulated PTH dynamics in patients with SHPT on cinacalcet across 4 baseline levels (300, 600, 800, 1200 pg/mL) (Fig. 2 and Table 2). For a baseline of 600 pg/mL, with an E_max_ of 290 pg/mL, PTH levels decreased to 420.6, 378.3, 348.5, and 330.7 pg/mL at 3, 6, 12, and 24 weeks, respectively. These levels represent reductions of 61.9%, 76.4%, 86.7%, and 92.9% of the E_max_, indicating that cinacalcet's effect on reducing PTH nears a plateau around 12 weeks.

**Table 2.** Distribution of typical efficacy values following cinacalcet administration among subjects at different covariate levels (median, 95% CI)

	3 weeks	6 weeks	12 weeks	24 weeks
Parathyroid hormone, pg/mL				
Baseline__PTH_ = 300	213.9 (186.6, 237.8)	193.6 (163.4, 221.4)	179.4 (147.9, 211.5)	170.9 (137.2, 203.9)
Baseline__PTH_ = 600	420.6 (384.1, 449.8)	378.3 (342.9, 409.1)	348.5 (314.6, 380.8)	330.7 (296.1, 364.5)
Baseline__PTH_ = 800	557.9 (514.4, 593.1)	501.1 (464.3, 536.2)	461.3 (425.5, 495.0)	437.2 (403.2, 471.2)
Baseline__PTH_ = 1200	833.1 (770.9, 881.1)	747.5 (698.7, 790.1)	687.1 (647.1, 725.5)	650.6 (614.8, 685.7)
Serum calcium (baseline = 9.7 mg/dl)				
VD used = 0%	8.54 (8.47, 8.62)	8.50 (8.44, 8.57)	8.50 (8.44, 8.57)	8.50 (8.44, 8.57)
VD used = 50%	8.83 (8.76, 8.90)	8.80 (8.73, 8.86)	8.80 (8.73, 8.86)	8.79 (8.73, 8.86)
VD used = 100%	9.11 (9.05, 9.18)	9.09 (9.03, 9.15)	9.09 (9.03, 9.15)	9.09 (9.03, 9.15)
Serum phosphorus (baseline = 5.785 mg/dL)				
-	5.353 (5.212, 5.494)	5.280 (5.137, 5.426)	5.261 (5.116, 5.403)	5.261 (5.114, 5.407)

At baseline PTH concentrations of 300, 600, 800, and 1200 pg/mL, PTH levels decreased by 86.1, 179.4, 242.1, and 366.9 pg/mL, respectively, by the third week. These reductions represent decreases of 28.7%, 29.9%, 30.3%, and 30.6% from baseline, aligning with or nearing the target reduction of 30%.

After 12 weeks of cinacalcet treatment, PTH levels decreased to 179.4, 348.8, 461.3, and 687.1 pg/mL for baseline concentrations of 300, 600, 800, and 1200 pg/mL, respectively. According to the KDIGO guideline recommended PTH target range of 130 to 600 pg/mL, patients with baseline PTH values of 1046 pg/mL or higher did not achieve the target after 12 weeks of treatment.

### Typical Effects of Serum Calcium at Different Proportions of Patients Using Vitamin D Compounds

Using the final model, we simulated serum calcium level changes over time for patients using vitamin D compounds at 0%, 50%, and 100%. For 50% usage, the E_max_ value was 0.91 mg/dL, with serum calcium decreases of 0.87, 0.90, 0.90, and 0.91 mg/dL at 3, 6, 12, and 24 weeks, respectively. These results indicate that cinacalcet's effect on reducing serum calcium reaches a plateau by 3 weeks.

After 12 weeks of cinacalcet use, serum calcium levels decreased by 1.20, 0.90, and 0.61 mg/dL for subjects using vitamin D compounds at proportions of 0%, 50%, and 100%, respectively. The ideal serum calcium range is 8.4 to 10 mg/dL. For subjects with a baseline value of 9.6 mg/dL not using vitamin D, serum calcium dropped to 8.4 mg/dL, the lower limit, after 12 weeks. Conversely, for a baseline of 10.6 mg/dL in subjects using vitamin D, levels remained above 10 mg/dL after 12 weeks.

### Typical Effects of Serum Phosphorus

No significant covariates affecting serum phosphorus were identified in this study. Simulations from the final model showed that the E_max_ for serum phosphorus is 0.524 mg/dL, with decreases of 0.432, 0.505, 0.524, and 0.524 mg/dL at 3, 6, 12, and 24 weeks, respectively, postcinacalcet administration. These results indicate that the maximum effect of cinacalcet on reducing serum phosphorus is achieved by 3 weeks.

### Other Potential Influencing Factors on the Efficacy of Cinacalcet

**Figure 3.**
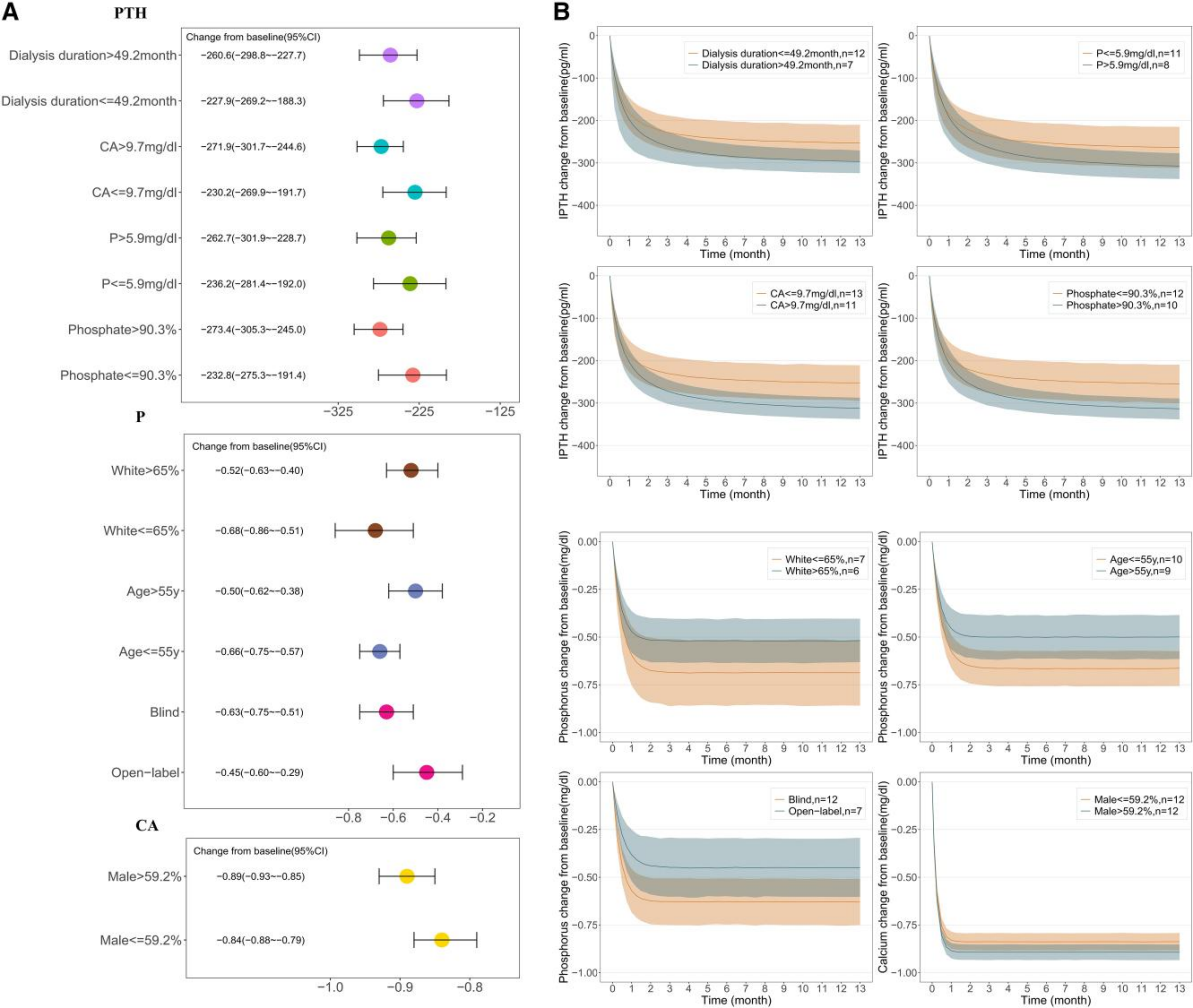
Subgroup analysis results indicative of trends affecting parathyroid hormone (PTH), serum calcium (CA), and serum phosphorus (P). (A) Each point and bar represent the typical value and the 95% CI for the change from baseline at 3 months in parathyroid hormone (PTH), serum calcium (CA), and serum phosphorus (P), respectively. (B) Solid lines denote the typical drug efficacy, while the shaded areas correspond to the 95% CI.

This study assessed additional factors influencing cinacalcet's efficacy through subgroup analysis, adjusting covariate levels to their median to mitigate heterogeneity effects. Findings indicate that factors affecting PTH response include dialysis duration, baseline serum calcium, baseline serum phosphorus, and the proportion of patients using phosphate binders (Fig. 3). Specifically, greater PTH reduction was noted with longer dialysis (>49.2 months), higher baseline serum calcium (>9.7 mg/dL), higher baseline serum phosphorus (>5.9 mg/dL), and higher usage of phosphate binders (>90.3%).

In the subgroup analysis examining serum calcium effects, a trend toward greater decreases were observed in trials with a high proportion of male patients (>59.2%). Factors potentially influencing serum phosphorus reduction included the proportion of White patients, trial blinding, and participant age. Specifically, greater decreases in serum phosphorus were noted when the proportion of White patients was below the median (65%), trials were blinded, and subjects were younger than the median age of 55 years.

We also conducted an exploratory analysis to investigate potential associations between the source of funding, risk of bias, and model parameters for PTH, calcium, serum calcium, and serum phosphorus, as detailed in Fig. S7 [17]. Our analysis did not reveal any significant associations among the model parameters, the source of funding, or the risk of bias.

### The Impact of Dosage on the Efficacy of Cinacalcet

**Figure 4.**
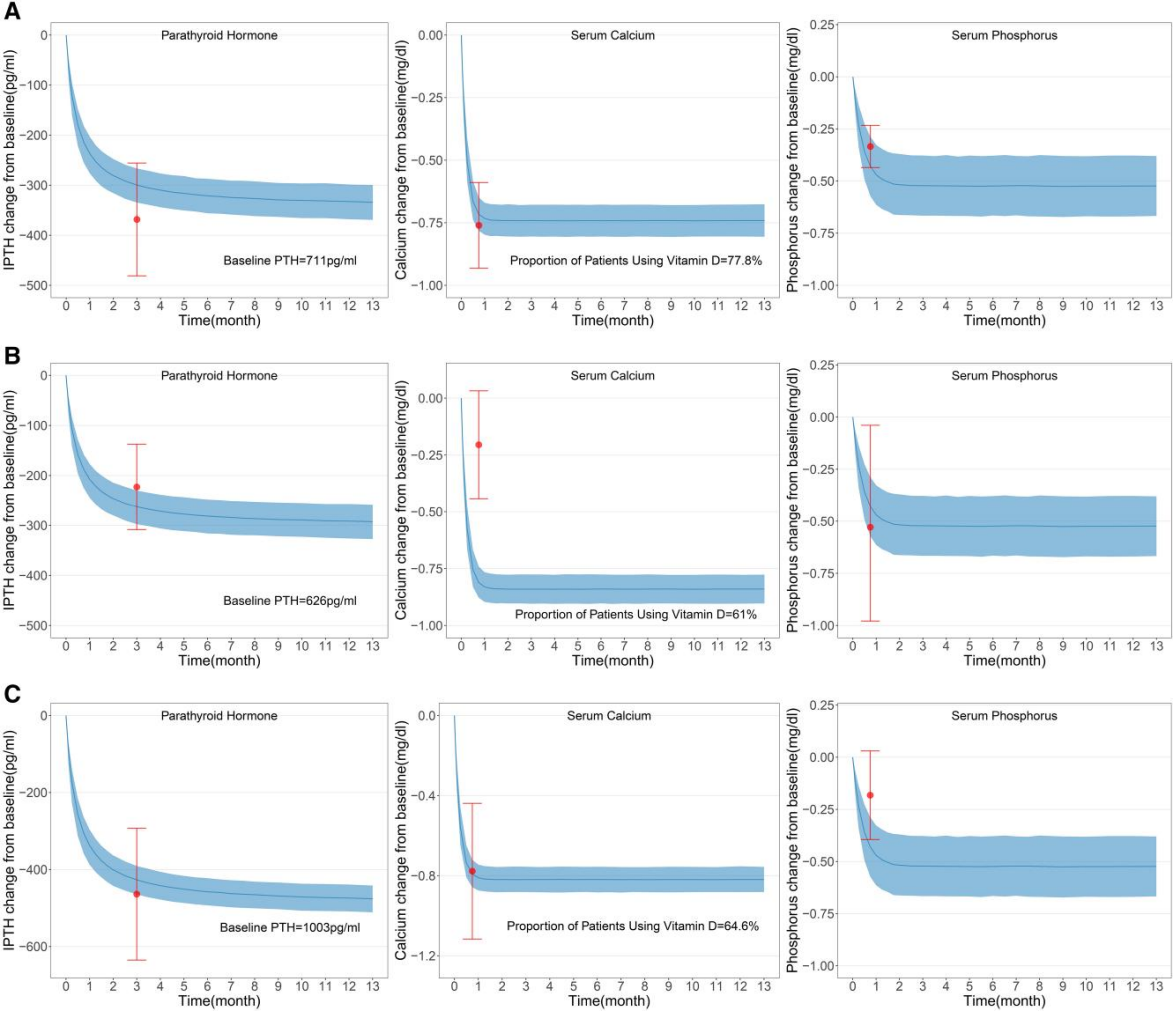
Comparison of typical efficacy within the 30 to 180 mg dose range of cinacalcet to measured efficacy within the 25 to 100 mg dose range. (A) Asian populations, (B) Western populations, and (C) the non-Japanese Asian population. The lines in the graph represent the predicted typical efficacy for the 30 to 180 mg dose range of cinacalcet at the same baseline as the measured efficacy for the 25 to 100 mg dose range, with the shaded areas indicating their 95% CI. The points in the graph denote the measured efficacy values for the 25 to 100 mg dose range of cinacalcet, and the error bars represent their 95% CI.

This study analyzed clinical trial data with cinacalcet dosages between 30 and 80 mg but excluded 5 Asian trials and 1 US/EU trial with dosages of 25 to 100 mg (Form S2 [17]). We simulated cinacalcet efficacy at 30 to 180 mg using baseline PTH levels and vitamin D usage from these 6 excluded trials as external controls for comparing efficacy against the 25 to 100 mg dosage (Fig. 4). Findings suggest that in the Asian population, efficacy at 25 to 100 mg is comparable to the 30 to 180 mg range. Moreover, the non-Japanese Asian population exhibited the same conclusions. However, in Western populations, the reduction in serum calcium at 25 to 100 mg was less than that observed in the 30 to 180 mg range.

## Discussion

Although cinacalcet's efficacy in reducing PTH is established, uncertainties remain regarding its performance across varying baseline PTH levels, achievement of target ranges, and time to optimal efficacy, which current guidelines do not fully address. This study, utilizing extensive literature, developed a clinical efficacy model for cinacalcet in treating SHPT and explored these issues. It found a significant correlation between the degree of PTH reduction and baseline PTH levels, with a 50.3 pg/mL increase in maximum PTH reduction for every 100 pg/mL increase in baseline PTH. However, higher baseline PTH levels led to substantial reductions but still resulted in elevated final PTH values. For instance, baseline PTH levels of 600 and 1200 pg/mL resulted in 12-week PTH levels of 348.5 and 687.1 pg/mL, respectively, with the latter significantly above the KDIGO recommended target range of 130 to 600 pg/mL. It was determined that achieving the target PTH level of below 600 pg/mL may be unfeasible for baseline PTH values exceeding 1046 pg/mL, suggesting the need for alternative strategies to lower PTH within the desired range.

Guidelines highlight the need to consider the interconnected changes in serum phosphorus, calcium, and PTH, and recommend that treatment strategies address these parameters comprehensively [4]. Post hoc analyses [19] indicate that cinacalcet treatment frequently results in hypocalcemia, with some patients experiencing serum calcium levels below the target range during monotherapy. Consequently, vitamin D is often coadministered in clinical settings to manage SHPT and elevate serum calcium levels. This study found a significant correlation between the degree of serum calcium reduction and the proportion of patients using vitamin D compounds. Specifically, serum calcium decreased by 1.20 mg/dL, 0.90 mg/dL, and 0.61 mg/dL when 0%, 50%, and 100% of patients used vitamin D compounds, respectively. Simulations indicate that for patients with baseline serum calcium below 9.6 mg/dL, combining vitamin D is advisable to prevent dropping to 8.4 mg/dL after 12 weeks. For those with baseline values above 10.6 mg/dL, adding vitamin D is unnecessary as levels remain above 10 mg/dL, indicating clear criteria for vitamin D coadministration.

Serum phosphate regulation is crucial in early SHPT management. This study demonstrates that cinacalcet can effectively lower serum phosphate levels, achieving a reduction of 0.432 mg/dL at 3 weeks, reaching a plateau of efficacy. The clinical studies analyzed in this article covered a range of subject serum phosphate levels, from 2.66 to 7.1 mg/dL. Within this range, no correlation was found between the reduction in serum phosphate and its baseline value.

According to cinacalcet's instructions for use [8], PTH concentration should be measured 1 to 4 weeks during dose titration to determine the need for adjustment. The efficacy model developed in this study accurately describes PTH changes following cinacalcet administration, identifying 3-week and 12-week post-treatment as critical monitoring points. PTH reduction reaches approximately 30% from baseline at 3 weeks and plateaus by 12 weeks. Monitoring at these times helps assess whether cinacalcet maintains normal PTH levels.

This study conducted a subgroup analysis to explore factors influencing cinacalcet's effectiveness beyond the identified covariates. It found that longer dialysis duration might enhance PTH reduction, and higher baseline serum calcium and phosphorus levels also showed greater PTH decrease, though these findings need further validation. Cinacalcet is widely used globally for SHPT management, with dosage variations across regions. Our analysis included 5 Asian studies, demonstrating that cinacalcet's efficacy at 25 to 100 mg in Asian patients is comparable to 30 to 180 mg in European and American patients. The Japanese Society for Dialysis Therapy recommends a PTH target of 60 to 240 pg/mL [20], lower than the KDIGO range. Despite this, lower doses in Japan effectively reduce PTH [21]. A Japanese clinical trial observed higher adverse reactions at a 50-mg dosage of cinacalcet compared with 12.5 and 25 mg, suggesting that lower doses may enhance safety. However, dose-dependent adverse events have not been observed in European and American populations [22]. Therefore, further data are needed to determine whether lower doses are also safer for European and American populations. High adherence to cinacalcet in Japan may be due to its safer profile at lower dosages, indicating that starting at 25 mg could provide a better benefit–risk balance. In Western populations, the reduction in serum calcium within the 25 to 100 mg dosage range was smaller than the 30 to 180 mg range, suggesting potential racial differences in the efficacy of cinacalcet. However, this study could only include 1 clinical trial from European and American populations using the 25 to 100 mg dosage for external comparison. Therefore, further research is necessary to confirm our findings regarding differences between Asian and White groups. Notably, existing studies exploring the influence of race and body weight on cinacalcet outcomes have primarily focused on White and African American populations [23].

This study faces several limitations. Research indicates that patients with SHPT often exhibit elevated fibroblast growth factor 23 levels, potentially linked to higher cardiovascular mortality, but data constraints precluded fibroblast growth factor 23 inclusion in our model [24]. Furthermore, over 30% missing data on critical factors like ethnicity hampered our analysis of these potential influences. Particularly regarding ethnicity, most studies have only reported the proportion of White participants without providing data on Asian populations. This omission precludes a thorough investigation into the impact of the proportion of Asian participants on the study outcomes. Beyond cinacalcet, calcimimetics such as evocalcet, etelcalcetide, and upacicalcet exist, yet scant clinical trial data restrict the development of a robust pharmacodynamic model to compare their efficacy [25]. Additionally, while dose titration is standard in cinacalcet administration, the lack of reported average doses in most trials prevented the construction of a precise dose–effect model, limiting us to broad comparisons across titration ranges. Finally, this study included only English-language publications, which may introduce publication bias.

### Conclusions

Based on comprehensive literature data, this study developed a pharmacodynamic model for cinacalcet treatment in SHPT. It predicts the time-course of efficacy indicators such as PTH, serum calcium, and serum phosphorus postadministration, along with covariate effects. This model aids in identifying suitable candidates for cinacalcet treatment and formulating optimal medication strategies, addressing key clinical considerations.

## Data Availability

The data that support the findings of this study are available from the corresponding author (Lujin Li), upon reasonable request. Supplemental material to this article can be found online at https://doi.org/10.5281/zenodo.14753993.

## Abbreviations

E_max_: maximum effects
KDIGO: Kidney Disease: Improving Global Outcomes
MBMA: model-based meta-analysis
PTH: parathyroid hormone
SHPT: secondary hyperparathyroidism
